# Game Theory in Mobile CrowdSensing: A Comprehensive Survey

**DOI:** 10.3390/s20072055

**Published:** 2020-04-06

**Authors:** Venkat Surya Dasari, Burak Kantarci, Maryam Pouryazdan, Luca Foschini, Michele Girolami

**Affiliations:** 1 School of Electrical Engineering and Computer Science, University of Ottawa, Ottawa, ON K1N 6N5, Canada; vdasa047@uottawa.ca; 2 Watts Water Technologies, North Andover, MA 01845, USA; maryam.pouryazdan@wattswater.com; 3 3 Department of Computer Science at the University of Bologna, 40136 Bologna, Italy; luca.foschini@unibo.it; 4 National Council of Research ISTI-CNR Italy, 56124 Pisa, Italy; michele.girolami@isti.cnr.it

**Keywords:** mobile crowdsensing, Internet of things, trustworthiness, user incentives, game theory

## Abstract

Mobile CrowdSensing (MCS) is an emerging paradigm in the distributed acquisition of smart city and Internet of Things (IoT) data. MCS requires large number of users to enable access to the built-in sensors in their mobile devices and share sensed data to ensure high value and high veracity of big sensed data. Improving user participation in MCS campaigns requires to boost users effectively, which is a key concern for the success of MCS platforms. As MCS builds on non-dedicated sensors, data trustworthiness cannot be guaranteed as every user attains an individual strategy to benefit from participation. At the same time, MCS platforms endeavor to acquire highly dependable crowd-sensed data at lower cost. This phenomenon introduces a game between users that form the participant pool, as well as between the participant pool and the MCS platform. Research on various game theoretic approaches aims to provide a stable solution to this problem. This article presents a comprehensive review of different game theoretic solutions that address the following issues in MCS such as sensing cost, quality of data, optimal price determination between data requesters and providers, and incentives. We propose a taxonomy of game theory-based solutions for MCS platforms in which problems are mainly formulated based on Stackelberg, Bayesian and Evolutionary games. We present the methods used by each game to reach an equilibrium where the solution for the problem ensures that every participant of the game is satisfied with their utility with no requirement of change in their strategies. The initial criterion to categorize the game theoretic solutions for MCS is based on co-operation and information available among participants whereas a participant could be either a requester or provider. Following a thorough qualitative comparison of the surveyed approaches, we provide insights concerning open areas and possible directions in this active field of research.

## 1. Introduction

**Figure 1:**
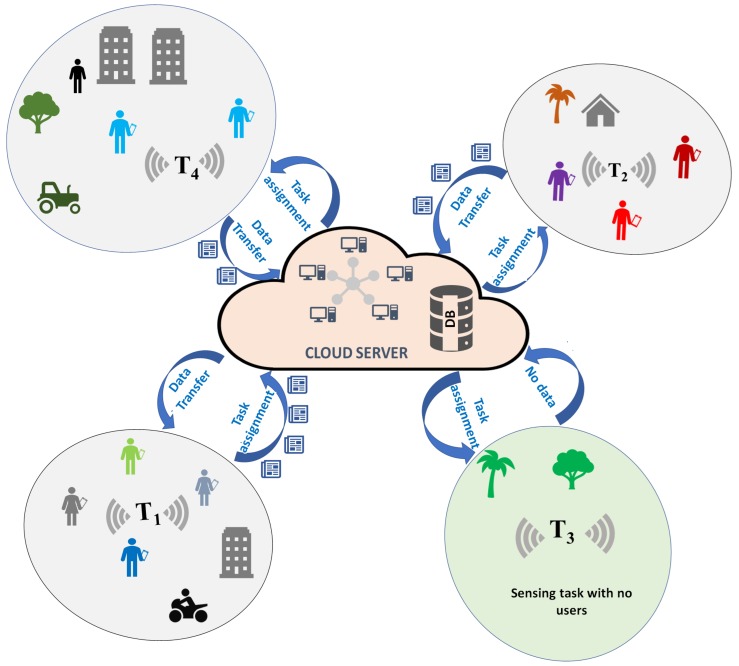
An illustration of data acquisition in a Mobile CrowdSensing (MCS) environment, border encompassing task $T_{i}$ denotes sensing task coverage. User with smart device in hand represents their preference to participate in sensing campaign.

With the massive deployment and wide adoption of the Internet of Things (IoT), the number of connected devices is tremendously increasing. This phenomenon enables monitoring and control of almost all physical infrastructures without dedicating significant amount of (fixed) sensing and computing resources. Sensed data plays a predominant role in IoT: According to Cisco, 2.5 quintillion bytes of daily data generation and 30 billion IoT-connected devices are forecast by the year 2020 [1]. Thus, it is vital to supplement the existing dedicated sensing infrastructures via sustainable and cost-efficient non-dedicated sensing solutions such as participatory or opportunistic sensing via smartphone sensors [2]. By taking advantage of embedded sensors in smart phones, sensed data can be collected in high volumes, and can be processed in real time in support of the IoT-based services. Widely known as Mobile CrowdSensing (MCS), this process is envisioned to be an integral component of IoT systems [3,4]. MCS empowers citizens with smart devices participate in the collection of sensed data by rewarding them for their contribution/effort. In MCS campaigns, sensed data is collected from various locations through built-in sensors of the mobile devices by either implicitly (opportunistic) or explicitly (participatory) recruiting users (see Figure 1). Acquired data is aggregated and processed, analyzed and visualized to support various services that lead to smart and sustainable spaces. As an example, a project by Microsoft, namely Nericell, aims to monitor traffic and driving conditions of roads through smart phone sensors [5]. In another project, called Mobile Century, GPS enabled mobile phones are recruited for traffic monitoring purposes [6]. Wide usage of social networking applications and skyrocketing number of mobile phones pave the way for MCS applications to be employed for various types of services such as quality of living, emergency preparedness, health care, smart transportation, environmental monitoring and public safety [7,8,9,10]. In Reference [11], an indoor application of MCS is considered where positioning and orientation information of landmark objects are estimated through mobile crowd-sensed data so to obtain indoor floor maps.

Despite the benefits listed above, the realization of robust MCS systems is still challenging. From the standpoint of an MCS platform, dependability/trustworthiness and veracity assurance of crowd-sensed data is a grand challenge as data acquisition involves anonymous and mainly unreliable sources. It is worth mentioning that lack of dependability or veracity of crowd-sensed data may occur due to various reasons such as intentional manipulation (i.e., malicious actions by adversaries) or malfunctioning of sensors, which might just be a temporary situation. Lack of ground truth at the MCS platform makes this problem further challenging. Nevertheless, to prevent MCS campaigns from vulnerabilities, effective user recruitment is the only tool that an MCS platform can use. More specifically, we consider that some possible solutions to prevent the lack of dependability [12,13,14] are: to design systems able to manage and update the reputation of the involved users by adopting optimal user’s recruiting policies; to dynamically assess and keep updated the trustworthiness of the involved users; and to adopt recruitment policies able to detect or event to predict the existence of non-cooperative users, that is, users not providing useful data for the MCS system. Reputation based selection schemes are widely used by researchers in such a way that reputation level became an added attribute/constraint for recruiting a participant [15,16,17,18,19].

From the standpoint of the participants, privacy is a primary concern that may de-incentivize users against participating in MCS campaigns. To overcome that potential issue and improve participation in sensing campaigns, related work proposes effective methods of incentivization, which can be in the form of either monetary or non-monetary (e.g., entertainment, badges, etc.) [20,21]. While the incentive’s being monetary or non-monetary is not within the scope of this study, it should be recognized that sensing, computing and transmission costs incurred during MCS campaigns call for compensation. Moreover, MCS requires diversity of participants in a campaign so as to have the fused sensory data as accurate as possible with respect to the actual value to increase the quality of crowdsensed data. Reddy et al. [22] examine participant behaviour in a range of monetary payoffs, and results indicate that provoking competition between participants may not help obtain high quality information through collaboratively sensed data. It is reported that proper design of micro-payments can lead the useful participation. A recent work introduced the concept of trading of tasks between users by considering the time required to tackle a task by a traded user [23]. Results supported the hypothesis that proposed mechanism could reduce total sensing time.

Finally, another important issue from the participant perspective is energy consumption. It is anticipated that in the near future it will be possible to power up smart devices through wireless signals. Vamsi et al. [24] were successful in delivering power to battery less temperature and camera sensors using Wi-Fi signals. This might extremely reduce the cost of sensing and facilitates data collection process by encouraging more number of users to participate in sensing campaigns without worry of battery. Moreover mobility of users in MCS improves sensing coverage area.

To tackle all above contrasting goals, several works in the recent literature are proposing to adopt a Game Theory approaches, where each participant aims to maximize its profit, namely the objective function of each player. Game theory analyzes the situation where multiple players are involved in, and a player’s gain does not only depend on their own strategy. In MCS, game can be formulated either among participants or between the platform and the participants. In the former case, participants of CrowdSensing compete among themselves to achieve higher profit and the decision of each participant will be influenced by the actions of others. In the latter case, decisions of the platform over user incentives are affected by participants’ strategies. In either case, the profit can apply in the form of maximizing the score, the reputation or the benefits obtained from users.

In this survey, we review various game theoretical methods applied in MCS that help to reach an equilibrium in a competition for gaining higher utility between service requester and service provider. We start with presenting a brief introduction to the typical game theory models. Next, we classify the game models applied in MCS on the basis of co-operation. This classification is further extended by the level of information, that is, complete or incomplete information, available among players involved in the formulated game. Finally, the paper presents open research areas that can be considered for future research in this field.

The rest of the paper is organized as follows. In Section 2, we give some needed background material about the MCS paradigm, while in Section 3 we introduce a general model for Game theory that can be applied to MCS focusing, especially, on some existing models for cooperative and non-cooperative games. We then present in Section 4 a possible adaptation of the Game theory model to the MCS paradigm by presenting a survey of existing works addressing such approach. In Section 5, we envision new directions and main open research areas in Game theory in MCS. Finally, Section 6 concludes the paper reporting final discussions.

## 2. Background and Challenges in Mobile Crowdsensing

Despite of potential benefits of MCS, few primary challenges must be considered for building a sustainable system: privacy and security, data quality, trustworthiness, energy consumption and incentives for participants. This section presents needed background material together with main emerging guidelines and solutions against those main five challenges under diverse scenarios.

### 2.1. Privacy and Security

Privacy is one of the most crucial issue that every participant is concerned with. For instance, information gathered from search history of an individual plays a crucial role in displaying advertisements in social networking applications. According to Hewlett Packard study there exist an average of 25 vulnerabilities that a device can be attacked through, and 70% of IoT devices are vulnerable to these attacks [25]. The shared data may contain sensitive information of mobile device as well as private information of users [26] which leads to privacy leaks. Access control should be permitted based on pre-established trust between user and service provider [27].

Moreover most of the MCS campaigns involve location-based tasks such as traffic congestion control, which reveal daily mobility patterns of users. Many location based services need to access the location of a user, Sastry et al. [28] proposed a simple technique to avoid malicious activities that utilize location based comforts without the physical presence. Most popular location spotting service is done by GPS (Global Positioning System) with the accuracy of less than 5 m, and can introduce vulnerabilities for attackers sending strong radio signals [29]. To illustrate potential privacy breaches, a route prediction model was proposed by Patterson et al. [30] based on historical behaviour of a user. It is worth noting that, contextual pattern analysis do not only reveal residential pattern by can lead to inferring the residential address of a user [31]. Yang et al. presented various privacy and security threats that might occur from crowd-sourced information. As a countermeasure, a CrowdSourcing architecture was proposed where task is fragmented before CrowdSourcing it [26].

In order to deal with disclosure of location information, the authors of Reference [32] proposed a model which distorts the precision of user location, and results show that privacy of user can be achieved with the cost of quality of data. In another work, Talasila et al. [33] proposed a framework to improve reliability of data based on location by eradicating malicious user with the help of Bluetooth discovery (i.e., every user should reside in range of each other to sense the same task). In an empirical study on human participants, the authors of Reference [34] found out that an obvious user discomfort in terms of privacy while majority of users seemed to prefer legal regulations due to lack of trust in human-initiated or automated mechanisms that claim to ensure privacy preservation. At this point, it is worth noting that there exists a trade-off between privacy preservation and data trustworthiness. Thus, obtaining trustworthy data from participant requires the crowd-sensed information to be analyzed by exploiting many features, which may cause de-incentivizing privacy concerns [35]. In order to cope with the requirements, Wang et al. [36] proposed a framework named ARTSense (Anonymity, Reputation, Trust) to preserve private data–particularly location and identity of users– while ensuring trustworthiness based on reputation of recruited users. On the other hand, the authors of References [37,38] argue that most of the users do not oppose to share their location.

### 2.2. Quality of Data

Quality of data is difficult to maintain in MCS due to various reasons for example, malfunctioning of sensors, environmental conditions. Low quality data costs hundreds of million USD loss only for US business as reported by the Data Warehousing Institute [39]. Moreover to obtain sensor data over a large area, high number of participants should be recruited; which comes at the expense of increased sensing budget for the CrowdSensing platform as the users expect incentives for providing sensing as a service. On the other hand data collected more than certain threshold may not help in the improvement of the data quality [40]. To overcome this problem, the authors of Reference [41] proposed an estimation strategy for the locations that cannot be covered by recruited participants. The proposed approach uses the neighbouring cell values however data obtained may not be accurate which may lead to the degradation of quality. The degradation of quality may result in poor decisions. The authors of Reference [42] showed that the higher the quality of data, the lesser the storage space required in the cloud. Liu et al. [43] proposed a mechanism in which a task is assigned to a group that has high credit index, and that can deliver the required quality denoted by the Quality of Information (QoI) satisfaction index.

Gong et al. [44] presented task assignment mechanisms under various scenarios to improve data quality and reported that the quality of data is associated to the task duration, data collection times and spatiotemporal coverage. On the other side missing data inference should be done in an error-free manner at the server to meet the quality requirements. Re-sampling techniques such as bootstrapping could be used to approximate the quality from sparse data to a reasonable value [45]. Kong et al. [46] used spatiotemporal correlations to deduce missing environmental data. Furthermore, the size of participant population can be reduced by selecting minimum number of cells to sense and pursuing inference of missing data [45]. Moreover, in the case of participatory sensing in MCS campaigns, data quality may vary due to lack of experience in participants (e.g., capturing blur photos, recording audio with microphone covered etc.). In addition to all, computational power and sensory capabilities of each smart phone varies, thus not all participants might be able to deliver the same level of quality of sensed data [47]. As most of the quality estimation mechanisms do not treat sensor error and user reliability differently, this naive assumption of not decoupling user and the smartphone in terms of reliability may lead to inaccurate evaluation of participants during the recruitment process. To address this challenge, the authors of Reference [48] proposed an algorithm that exploits the confusion matrix and separates user and device errors to quantify the quality of a participant.

### 2.3. Trustworthiness

Trustworthiness plays a vital role in participatory sensing, since data obtained from participants influence decision making. Trust can be established in the form of either trust by reliability or trust by decision [49]. The former is computed on an entity, and denotes the probability by which that entity reports truthfully whereas the latter stands for the situation where an entity is trusted irrespective of its possible errors. For example trust on anonymous participants could be considered as reliability trust whereas trust on anchored sources/dedicated sensors would be analogous to decision trust. For this reason MCS needs more reliable trusted participants to pursue an action since it is based on non-dedicated sensors, and the acquired data can be used for the development of critical CrowdSensing applications such as health care [50], and pollution monitoring [51]. Sybil attacks are also possible in MCS. Chang et al. [52] proposed a framework which detects changes towards heavy traffic intensity through active and passive tests so as to conduct trust credit assessment based on previous records to recommend whether or not the traffic intensity is injected by Sybil nodes. Another issue in CrowdSensing is the level of reliability on security mechanisms, that is, to which extent can the security mechanism be trusted? Huang et al. used Gompertz function to design a reputation system that ensures trustworthiness in the data received [53]. Pouryazdan et al. [54] presented a detailed comparison among various trustworthy mechanisms where users are assumed to be static (i.e., not mobile). One of the other proposed approaches to tackle trustworthiness is the use of collaborative reputation scores that is, the combination of statistical reputation [55] as well as vote-based reputation score [56] along with anchor assistance. Chen et al. [35] proposed supervised and unsupervised trust weight calculation methods for crowdsourcing, which could also be adopted by MCS systems. Another work related to trustworthiness [48] shows the proposed model ’mPASS’ could perform effectively even under malicious environments. Reliability/trustworthiness was evaluated by analyzing the credibility of a user with sensor accuracy. Wengdong et al focused on considering attentiveness/interest of user along with quality of the data submitted, in order to evaluate the most trusted user. Attentiveness/willingness is defined as the shortest time window in which a user joins an MCS campaign to undertake sensing tasks [57]. On the other hand, a participant may not exhibit the same level of effectiveness in all types of tasks. That being said, it is possible to experience situations when recruitment of a highly reputed participant might be inadequate. Amintoos and Kanhere considered this challenge and calculated various reputation based parameters such as quality of data, region of interest, field of expertize and also response time. Their simulation results showed 15% increase of trust in proposed scheme when compared to baseline mechanisms [58].

### 2.4. Energy

Battery is the source of energy, driving force of a mobile device. Regulation of energy consumption has almost equal concern in MCS with the protection of security and privacy of users. Sensing, raw data processing and transferring of collected data require battery power. Battery level reduction of a device depends on many factors such as Operating System (OS) used by the device, type and number of sensors used for sensing and moreover GPS consumes different amount of energy whether device is indoor or outdoor [59]. Energy consumption and rate of data transfer varies depending on the type of network mode we choose (3G, WiFi, LTE, etc.) [60] and sampling frequency [61]. Liu et al. [43] proposed a distributed energy management scheme in which users are penalized for using high energy even though they might be maintaining the quality of data.

Anjomshoa et al. [62] considered residual battery level of devices in user recruitment since low residual energy is one of the reason for opting out from a sensing campaign. In Reference [63], Liu et al. claimed that region of interest change from time to time and there may be a case where the platform cannot find users having sufficient battery life, which would eventually result in low Quality of Information (QoI). In order to acquire better QoI in the aforementioned case, an energy aware selection model was proposed where users’ sampling rate varied based on battery level so as to enable the platform to recruit low-battery users to meet the required quality of information. The authors of Reference [64] dealt with network protocols to prevail energy efficient transmission with the deployment of static nodes in sensing environment.

Tomasoni et al. [65] analyzed energy conserving capabilities of different data collection frameworks and claimed that energy consumption during reporting of data is higher compared to sensing of data. As reported by Tomasoni et al., battery draining occurs not only while sensing and transferring data but also while waking up the phone so Lane et al. [66] proposed the Piggyback CrowdSensing (PCS) to overcome this challenge. In PCS, sensing begins when other applications such as location detection during operation of Google maps or phone calls to avoid energy overhead due to waking up the device, which consumes considerable amount of energy at high sensing cost.

### 2.5. Incentives

CrowdSensing participants utilize their own resources to sense and send the required information. It is obvious that no participant wish to sacrifice their time, energy and cost without receiving any benefit. The system should be designed to motivate crowd to participate by offering incentives. Moreover design of an incentive mechanism in any CrowdSensing environment is crucial because of fixed budgets in most of the MCS platforms. Every user in system should not be incentivized fairly as quality of service or contribution provided to the task varies from user to user. The following question remains—How could one quantify the quality of data received from a user without any ground truth? The authors of Reference [67] estimated quality of data and level of contribution using information theory on top of Expectation Maximization (EM) algorithm. Some tasks do not compromise in data quality, other may need more coverage area. Effective incentive mechanisms can tackle this situation up to a great extent. In case of data quality, incentivizing users based on their reputation might be a possible approach whereas in the case of large coverage, availability of user is of great concern. To attract users to a sensing location, incentives offered should not be less than the actual sensing costs, this price imbalance is one of the challenges presented in a detailed survey of incentive mechanisms in MCS [68]. To address this situation, recruiting participants based on mobility patterns is proposed by Reddy et al. [69]. One of the popular approaches to recruit participants for lower budget is through reverse auction. Participants tend to drop from sensing campaign because of not being selected. Lee et al. [70] proposed a participant virtual credit concept in support of non-winners so that they have high chances of winning (i.e., being selected) in future rounds.

Offline incentive mechanisms may not work in real time as they assume user could wait until the completion of biding by other users. To overcome this challenge, the authors of References [71,72] proposed an online incentive mechanism. As most of the users are busy in rush hours, participants for sensing campaign may not be sufficient which increases dependence on available participants. An attempt to attract users in aforementioned case, Wang et al. [73] proposed a two level heterogeneous incentive mechanism by allocating rewards based on spatio-temporal popularity of certain task.

### 2.6. Agent-Based Strategies

Besides leveraging user-provided data, it is also imperative to incorporate agents with MCS campaigns [74]. Normally, mobile agents would be deployed in a crowdsensing setting to collect and/or query data [75] or perform other tasks as overviewed below.

With the advent of augmented and virtual reality communications, the authors of Reference [76] propose to consolidate an agent-based approach with crowdsensing campaigns so to enable virtual and augmented reality in a MCS setting.

Leveraging agents is also considered to cope with the untrusted nature of MCS servers. With this in mind, the authors of Reference [77] propose a distributed agent-based strategy to ensure a certain level of privacy where crowd-sourced data goes through every agent following perturbation of aggregated statistics at the agents.

## 3. Presentation of Common Game Theory Models

This section provides an overview of main Game Theory approaches, by also elaborating on the possible advantages of their application in the MCS context and introducing the main taxonomy directions used to analyze all proposals surveyed in the next section.

Game theory has been a useful tool to model the behavior of participants and platforms to maximize their utilities [78]. Primary reason behind formulating these interactions in the format of games stems from the selfishness and rationality of participants. Thus, in the existence of selfish and rational users, cooperativeness of the participants can be ensured through effective games [79]. A typical use case that leverage game theory is the design of incentives to recruit a sufficient size of participant population for the sensing campaigns [80]. In addition, pricing strategies for the value of the data so to eliminate untrutful participants in MCS campaigns [81] is to address the security and trustworthiness concerns in MCS campaigns. As mentioned earlier, energy consumption is one of the roadblocks against wide adoption of MCS. With this in mind, game theory serves as a useful tool to make a reasonable compromise between the utility of the MCS platform (i.e., delivery ratio of crowd-sensed data) and energy consumption of participating devices [82]. Another challenge for MCS systems is the privacy concerns of participants. To this end, games need to be formulated between participants and the MCS platform in order to maximize the privacy requirements of the participants while maximizing the utility of the platform [83].

**Figure 2:**
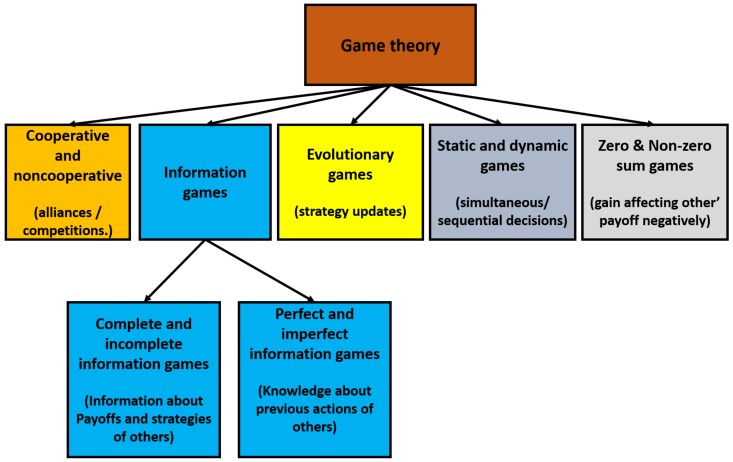
Classification of game theoretic models.

The three major components that make up the game models are players, strategies and payoffs. A player denotes a decision making entity whereas a strategy is analogous to a set of rules for decision under various situations. Payoff can be either a reward or loss that players experience when they implement their respective strategies. Figure 2 presents the popular models in game theoretic solutions. Game theoretic solutions are examined under five categories: co-operative/non-co-operative games, information games, evolutionary games, static/dynamic games, and zero/non-zero games. Among these five categories, information games can further be broken down into two sub-categories: complete/incomplete information games and perfect/imperfect information games. In the following, we decided to organize the section structure according to the above categorization; hence, each subsection will detail a category, and we further split the information games subsection into two parts.

### 3.1. Co-Operative and Non-Co-Operative Games

The primary criterion for the classification of games is based on the co-operation between players. Winning strategy of a player varies from one game to another. When users co-operate with each other in order to receive a better payoff, a co-operative game is formulated. In a co-operative game, players receive assistance from third parties in order to enable co-operation ensuring through sanctions and incentives between players instead of defecting others. This is to avoid any dishonesty in participation. Co-operative game assumes that participants can achieve certain outcomes among themselves through co-operative agreements. Participants/players aim at common goals and interests that enable them to establish trust and co-operate. Co-operation will be successful when payoff or gain obtained by co-operation is considerably higher than the individual efforts of the players. In mobile CrowdSensing campaigns, participants are encouraged/incentivized to co-operate by the data collector/platform so as to maximize their utility [84]. One way to impose co-operation in the prisoner’s dilemma is to repeat the game [85] which results in better equilibrium than competing with each other.

On the other hand, a game is said to be non-co-operative if players cannot form clusters required to enable co-operation. Non-co-operative game treats each participant as a unit of analysis as it assumes each participant acts with respect to their self interest whereas participants of a co-operative game form groups to obtain better payoffs, and the unit of analysis is more often a group/subgroup of participants.

### 3.2. Information Games

#### 3.2.1. Perfect and Imperfect Information Games

In games with perfect information, participants have knowledge about previous decisions made by other participants of a game [86]. Tic-tac-toe and ultimatum games are possible examples of perfect information games. In an ultimatum game [87], one of the players (say player 1) receives payment and proposes a split ratio to another player involved (say player 2). Player 2 can choose to accept or reject the proposed offer, and if rejected by player 2, none of the players receives the payment. In perfect information games only one player moves at each timestamp by observing the decision of the other player. Chess can be one of the popular example for perfect information game. In the mobile networks context, with the help of the perfect information games, the problem of having selfish users in multi-packet slotted aloha was analyzed in Reference [88], whereas an imperfect information game would constitute common knowledge of possible strategies and type of the player but not the actions opted by others.

#### 3.2.2. Complete and Incomplete Information Games

As stated earlier, information is the major source of reference while making a decision. Participants of games with complete information can be equipped with the knowledge of possible strategies and the payoffs of others. Unlike the complete information games, in incomplete information games, payoffs and strategies of other participants are not completely known. Incomplete information games can be further classified as symmetric and asymmetric. Asymmetric incomplete information is a case where every player has some private information which is unaware of others. In a game of asymmetric information, a player’s gain mainly depends on amount of information possessed by the player [89].

### 3.3. Evolutionary Games

Evolutionary games are highly competitive where players can update their strategies to increase their gain/payoff. A repeated game does not guarantee that a winning strategy remain winning. For example in classical game theory either defectors (in a non-co-operative game ) or co-operators (in co-operative game) will get higher payoff. But in the case of evolutionary games, this phenomenon may vary from time to time. The change in strategies may sound evolutionary games as unstable. There can be an evolutionary stable strategy when adopted by almost every member of the group, and no other external person can disrupt the system with a new strategy. It might be possible to predict user behavior when a player repeats his/her set of strategies [90]. Repeated game falls under category of evolutionary games. Maintaining good reputation may be an asset and helps to get better payoff under repeated games [91].

### 3.4. Static and Dynamic Games

Games are typically represented in either normal or extensive form [92]. Games in which players make decisions simultaneously without knowledge of others decisions are known as static games. Prisoners dilemma, sealed auction bid are some of the examples of a static game [93]. This form is comprised of list of participants, list of strategies for each player and payoffs for each participant and represented in matrix as follows.

In a dynamic game, choices are made sequentially over time, or game is repeated [93]. Best example to illustrate dynamic game is the checkers game where each player have multiple possibilities to make a move based on the previous move of an opponent [94]. Players engaged in a sequential game make a move by anticipating the final outcome, that is, prefer to obtain final long term gain rather than short term. Participants in a game make decisions based on the information available to them.

### 3.5. Zero Sum and Non Zero-Sum Games

Zero-sum games are one of the cases in constant sum games, where gain of one participant has a negative effect on other participants’ payoff [95]. At any point of the game, the overall gain of participants is always summed up to zero whereas in constant sum game, it is a fixed value. Since each participant in zero-sum games completely oppose others’ interests, there is no scope of collaborative strategies, moreover it lists under strictly competitive games. Most of the sporting games come under strictly competitive in zero-sum games. Some of the classic examples of zero sum game are chess followed by boxing and so forth. In contrast, if profit and loss of all players do not add up to zero/constant sum, then it ends up being a non-zero sum game. Here, participants strategies are neither collaborative nor completely opposite, also called mixed strategies that is, participants partially compete with each other and partially co-operate for better payoffs. In mixed strategy games, one participants gain does not necessarily result in others’ loss [96].

## 4. Game Theory in MCS

**Figure 3:**
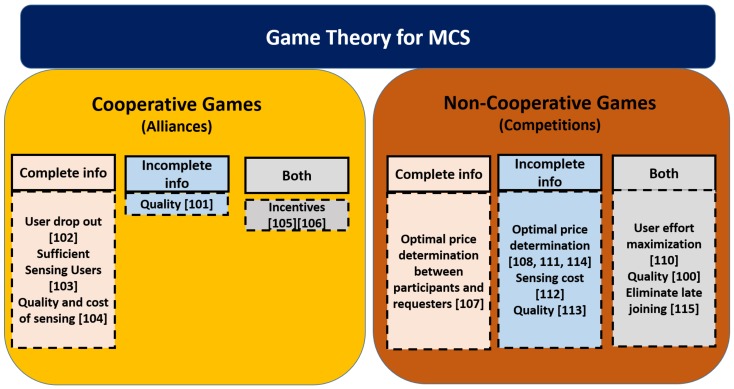
Main issues addressed by surveyed solutions according to the game theoretic approaches.

This section proceeds by stating the motivation of using Game Theory in MCS followed by subsections that present surveyed works dividing the in those adopting co-operative and non-co-operative game approaches under complete and incomplete information. Let us also anticipate that Table 1 will provide and outline pros and cons of each surveyed solution with the type of game used. Figure 3, instead, synthesize all main issues addressed by surveyed solutions according to the game theoretic approaches.

The motivation in applying game theory in MCS is to cope with the competitive behavior exhibited by participants to get higher utilities/benefits. Data requester aims to get high quality, trustworthy data within low budget whereas users aim to be compensated for their sensing service. Moreover, gain and loss in opportunistic sensing does not entirely depend on oneself rather it also relies on other participants’ strategies and moves. Hence game theory could be beneficial in order to analyze this competitiveness among sensing participants, sensing requesters.

CrowdSourcing takes advantage of peoples interest in playing games and formulates games through which required information is acquired. Dion et al. investigated the impact of a virtual reward on data quality and satisfaction of the user [112]. Three types of virtual rewards (badges, points and no reward) were considered. Results show that users are well motivated when rewarded through badges and moreover quality of data has improved due to virtual rewards in crowdsourcing.

Cyber physical social systems (CPSS) play a crucial role in improving the quality of life, some of the prominent examples of cyber physical systems are smart grids, autonomous vehicles and medical monitoring. With this in mind, the authors of Reference [113] took a step forward and introduced an incentive mechanism for improvement in CrowdSourcing for CPSS, where users were classified under three categories (malicious, speculative and honest users), and an auction game was modeled where compensation was provided based on user reputation while users aim to reduce their sensing costs in order for them to be selected. Authors assume that faults from sensors are negligible.

Zhi-Gang et al. focused on the impact of game history on users’ preference to co-operate in a game [84]. In evolutionary games, participants study strategies of predecessor and modify their decisions for the next round of game. Indeed, formulating an evolutionary game is at the expense of extra memory usage overhead. In order to investigate the memory effect on co-operation, authors consider three different dilemma games (Prisoner’s Dilemma, Snowdrift game and Stag Hunt game ) and make the following conclusion. The users/participants with memory unconditionally follow the strategy of a participant whose payoff is higher than his/her own, and posses less co-operation ratio compared to the agents whose history is randomly initialized.

As massive connectivity will leverage the concept of Industrial Internet of Things, Industry 4.0 can also leverage the concept of mobile CrowdSensing as stated in Reference [114]. Since users are eager to increase their payoff data quality and overall performance would be demolished. To maintain social welfare in games between requester and sensing user, the authors of Reference [115] proposed a zero-determinant strategy to introduce a linear relationship between co-players’ pay-offs without them considering each other’s strategy. The results show that the proposal outperforms co-operative, defective and random strategies adopted by the requester.

### 4.1. Co-Operative Games in MCS

**Figure 4:**
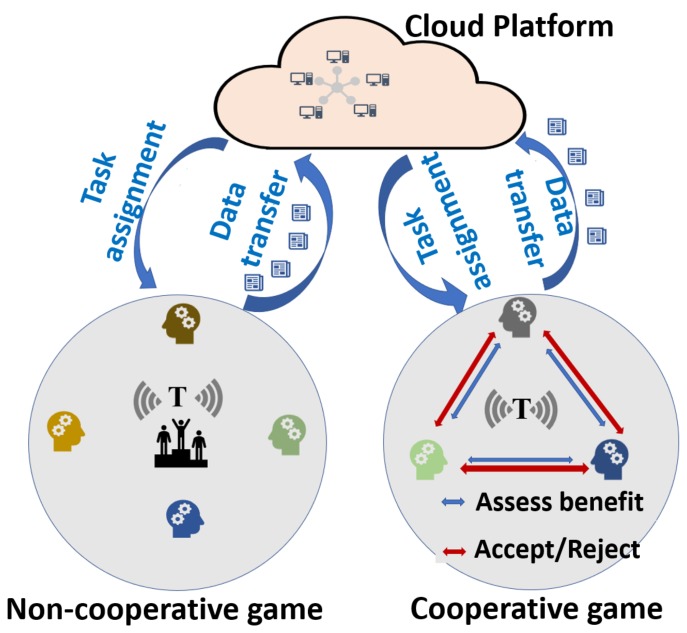
Figure describing types of co-operation among participants.

Co-operation among players of game can happen only if both players believe in benefit of collaborating with each other, Figure 4 depicts the same. Here information among players might be sensing cost, quality of data, user biding, payoffs, strategies and so forth, depending the context. Co-operation can occur between users or between data requesters and users since all the participants who are involved in MCS are parts of the game. Co-operation between requester and user leads to flexibility in the budget resulting in veracity of the data obtained [100]. In addition, configuring co-operative games in MCS helps in maintaining the balance in the number of participants [99,108]. A detailed and synthetic view of all solutions surveyed in the following is available in Table 1 and Figure 3.

#### 4.1.1. Co-Operative Games in MCS with Complete Information

Jaimes et al. [108] study the problem of participants dropping out from sensing system when the platform persistently excludes them from sensing campaigns. This is not a desired situation for the system since it is preferred to have more number of participants for high value in the crowd-sensed data. To overcome this issue, a co-operative incentive mechanism was introduced in which former winner tries to share some of their profit with neighbors in order to keep their bids higher and as a consequence making the winner win again. If one neighbour rejects the offer, the winner reduces their bid however rejection can happen if and only if the minimum profit of user *i* is greater than the proposed offer or at least one neighbour’s bid is less than the winner’s bid. Profit is shared among the winner’s neighbours based on their bids. Here, participants co-operate rather than competing with each other so that each of them can benefit from participating in the game. However this approach can be limited to only repeated games.

In co-operative spectrum sensing secondary users try to be rational and do not join co-operative sensing campaign unless motivated by some incentives. To address this issue, the authors of Reference [99] proposed a co-operative game based time optimization model where each player alter their own time strategy to maximize co-operative sensing and users are recruited based on their current reputation value.

Yang et al. [111] proposed a social incentive mechanism by promoting co-operation between users for better quality of information. A three stage Stackelberg game is designed where in additional stage, players exert pressure on their friends based on difference between current strategy and strategy at Nash equilibrium to get high payoff. Also proved that proposed social co-operative mechanism is cost effective where decrease in quality of information effects everyone since gathered information is shared by everyone, e.g., Live traffic updates.

#### 4.1.2. Co-Operative Games in MCS with Incomplete Information

In order to improve data quality in mobile CrowdSensing Yang et al. [100] proposed a quality estimation model through unsupervised learning followed by surplus sharing module designed as a co-operative game between users and platform where payments for users are calculated based on Shapley value and. The main motivation here is platform does not have fixed budget but surplus earned by platform depend on users quality of contributions. In oder to evaluate data trustworthiness authors proposed a truth estimation model that performed better compared with other heuristic models. Moreover they assume quality data providers dominate at the start of truth estimation model and all users have homogeneous devices. Formulation of co-operative game between platform and users helps for realistic dynamic flow budget.

#### 4.1.3. Co-Operative Games in MCS Considering Both Complete and Incomplete Information

Luo et al. [106] state that traditional incentive mechanisms have not taken into account users performing in diverse multiple tasks according to priority of task. Four incentive mechanisms were introduced with the help of stackelberg game based on information( complete and incomplete) of user and type of tasks (homogeneous or heterogeneous). They took a step ahead and calculated time complexity for their proposed algorithms. Results show that reward function based on number of users favors the platform, whereas rewards based on the value of a task is profitable for users.

Duan et al. [107] focused on with incentive mechanisms for both acquisition of data and for distributed computing. They assumed that users are homogeneous in reporting data but differ in their computational power then considered two cases (i) complete information/symmetrically incomplete information( knowledge about cost of sensing is same for client and user), (ii) asymmetrical incomplete information (only client is unaware of sensing cost). They formulated reward based incentive mechanism for case-(i) with the help of the two stage Stackelberg game, where in stage-1 client advertise total reward and minimum number of collaborators and users under a game probably reach Nash equilibrium. Besides that contract based incentive mechanism was used for case-(ii) to incentivize users based on their efficiency in which contract is proposed by client and users accept contract only if it is profitable.

### 4.2. Non-Co-Operative Games in MCS

Non-co-operation among players appear when co-operation does not yield to maximum payoffs in a competitive games. Co-operation among players may not solve optimal price determination [102] for data collected, that satisfy both user and requester. Non- co-operation among users indirectly compel users to provide high quality data [103,109], improving the effort of users [101]. Researchers determine solutions from Equilibrium obtained from Non-co-operative game.

#### 4.2.1. Non-Co-Operative Games in MCS with Complete Information

The authors of Reference [102] took advantage of concept of peer to peer communication to overshadow the draw back of high cost and poor adaptability of traditional centralized server with increase in number of tasks. User store the sensed data and share among themselves for a reasonable incentive, assuming that different tasks have same size of data interpreting that upload and download costs are almost same for every user which may not be true in practical scenario. Since every user would like to get maximum benefit in every transaction they formulated a non-co-operative game using Wardrop equilibrium (notation of Nash equilibrium for games over networks) and user payoffs are determined based on quality, revenue sharing scheme.

#### 4.2.2. Non-Co-Operative Games in MCS with Incomplete Information

Peng et al. [105] anticipate the possibility of competition between crowd sourcers and proposed an evolutionary based algorithm where users choose a crowd sourcer dynamically to sense and share data. Accordingly if the payment received is less than the average payoff by that particular crowd sourcer, user selects another probable crowd sourcer until he settle. Where in case of crowd sourcers rivalry in process of attracting users, they tend to increase the budget which leads to decrease in profit. Authors treated this rivalry as non-co-operative game and solution is presented as Unique Nash equilibrium, where every crowd sourcer have similar budget and equal distribution of users among themselves.

Former incentive mechanisms haven’t took a detailed look into the cost of wifi and its availability in order to provide adequate incentives to the users. Data providers( users) using mobile data have different sensing cost and coverage area compared to Wi-Fi users, which are to be considered to make an effective payoff. Cheung et al. [104] introduced delay sensitive MCS by formulating a two stage stackelberg game to model interactions between platform and user. However user recruitment strategies are not clearly mentioned and it is hard to platform to have complete information of wifi availability and costs for each and every user in real scenarios.

Usually, sensing campaigns contain diverse users with enclosed personal information (for example, sensing cost). The intuitive game formed is, platform would try to increase its utility by recruiting users for cheaper price if complete information of user is known, but users make sure that it is confined. The authors of Reference [97] analysed this challenge and came with an optimal solution where users payoff is determined by his amount of contribution that helps in increase of platform /cloud sourcer utility and rewards are based on prize tuple. They also mention that heterogeneous users with incomplete information behave like if they have homogeneous setting that is, individually rational.

Another incentive mechanism was proposed by Xie et al. [98] based on requesters rating on received data. They formulated reputation protocol which eliminates users by considering history of low quality data. In order to quantify the minimum payoff offered to the high quality data, authors used repeated game to characterize users behaviour by enclosing users skill as a private information. Effect of requesters bias in rating the received data was mentioned as future work.

Dong et al. [109] subdivided crowd sourcer into sensing platform, task originator considering that both are taken care by different companies in real world. Authors represented the competition between multiple platforms as dynamic non-co-operative game. Strategies of other players in this game are unknown, each platform resolve to a pricing strategy based on response of other platforms and task originators. The interaction between platform and task originator is formulated as multi leader stackelberg game (since there exist multiple platforms). At first platforms propose the price and then task originator have a strategy to maximize its utility by opting the optimal platform, based on quality of service. The solution is given as Nash equilibrium at which no player can improve his utility by changing his strategy alone and is determined by the proposed iterative algorithm.

#### 4.2.3. Non-Coperative Games in MCS Considering Both Complete and Incomplete Information

Jin in Reference [101] introduce an incentive mechanism named Theseus which generates a non-co-operative game where users opt different level of effort in sensing. User payoffs are based on their quality of data which is paramaterized by error probability (calculated from truth discovery algorithm). By this they assure that at Bayesian Nash Equilibrium all users try to utilize their maximum efforts to provide high quality data. Many incentive mechanisms were proposed in CrowdSensing, out of which contract based model is one of them.

Li et al. [103] designed two different quality aware incentive mechanisms for full information(QUAC-F) and incomplete information(QUAC-I). In case of full information ( cost function, ability distribution and risk attitude, etc.) stackelberg game was induced in which platform is leader and a contract based approach for incomplete information.

Xu et al. [110] proposed an incentive mechanism based on the time stamp of joining in sensing task. Motive behind this model is to receive more efforts from the early user by providing better incentives to the early user. They formulated two games one with complete information and other is incomplete (i.e., contributors joining time is a private information). In order to demonstrate the competition between users, stackelberg bayesian game is designed and incentive mechanisms were introduced to show the disparity between early and late users with unique bayesian equilibrium for each mechanism.

## 5. Open Research Areas

This section, without any pretence of being exhaustive, highlights open research areas that we deem crucial to address still open issues in applying game theory to MCS. In particular, the following section covers five possible research areas in the field of game theory, namely Evolutionary games, Nesh Equilibrium, Stackelberg Game, Co-operative games and Non-cooperative games.

### 5.1. Evolutionary Games

Reaching to an equilibrium state in evolutionary games may take too long as strategies of user change dynamically with the outcome of every iteration until they get a satisfied payoff. This nature of game may sometimes leads to unstable system. It is important to consider the time taken to acquire stability while designing game. Outcome of game could be unpredictable due to changing strategies of rational users. Opted strategy should be robust such that it can compete with itself along with succeeding against others strategy [116].

### 5.2. Nash Equilibrium

Game theory assumes users always behave rationally, each and every individual is sufficiently intelligent to take an optimal self-interested decision. This assumption may be impractical in CrowdSensing, as it deals with several human beings each of them having different level of thinking ability. For instance most of the users would like to adopt best strategy whereas some greedy, naive users may be inclined to risk so there would be a chance of getting higher payoffs, which leads to unpredictable equilibrium.

### 5.3. Stackelberg Game

Most of the non-co-operative games in CrowdSensing have incorporated stackelberg game. Similar to other games stackelberg game also have limitation of assuming that players behave in bounded rationality. Moreover it assumes that players can perfectly observe leaders strategy, which may not be true in dealing with humans [117] due to difference in abilities to observe. Followers deviation from optimal strategy could degrade utility of platform. In addition to that [118], assume that players opt a strategy which favours the platform when tie between strategies appear. At equilibrium follower could try to understand, decrypt the strategy of leader [119], which leads to loss for leader in following game.

### 5.4. Co-Operative Games

Enabling complete information within users in a co-operative game may lead to loss of platform utility. An appropriate example of this scenario could be [108] where in a reverse auction, neighbours bidding information is accessible to users. This may lead to combined attack strategy by users in desire of receiving higher rewards. However complete information with co-operation would be fruitful if game is between people who are familiar with each other. Most of the sensing participants may not be comfortable to co-operate with strangers. On the other hand user may not be completely sure of benefit from collaboration because of dubious rationality of others.

### 5.5. Non-Cooperative Games

One of the major barrier for building non-co-operative games in MCS is excessive competition with non-co-operation may lead to destruction in motivation of user to participate in sensing campaigns. This effects availability of sensing users for CrowdSensing. Furthermore the trade-off between individual benefit( by non-co-operation) of user and collaboration benefit should be addressed because of various influential factors that effect rational thinking.

## 6. Conclusions and Discussion

Mobile CrowdSensing (MCS) has recently become an emerging paradigm for large-scale distributed sensing campaign. MCS takes advantages of several advancements in the field of sensing devices, network capabilities, communication infrastructures and data analytic knowledge. This survey focuses on the adoption of the Game theory in order to model how users can be involved into the MCS loop. Game theory provides interesting comparison between a player and a data provider. Users are players whose goal is to maximize a possible utility function by performing actions that benefit the MCS campaign and, at the same time, increase this function. Of course, the Game theory approach can fail in predicting the human behaviour since he/she may suddenly act in different way irrespective of the better outcome. Moreover, players might fail to apply the best of all possible strategies to get higher payoff, this happens since a sensing user (ordinary people) may not understand game, as a result a platform may lose a high trustworthy user. Apart from exceptions, Game theory provides a better solution in a self-intended competitive environment. Furthermore, co-operation could not be a better option for tasks which require data instantaneously without any delay since establishment of co-operation between user might cause delay.

The combination of a Game theory with the MCS paradigm represents a promising approach for increasing the effectiveness of the data collection campaign. However, we consider that some existing barriers still represent the major barrier for a massive recruitment of uses. As a meaningful example, we consider issues related to the management of personal information and how such data are preserved by the MCS organizers. Moreover, the user of a MCS mobile app for collecting data might induce scepticism from the end-users by limiting their adoption. Finally, we consider that users have to be aware of the benefit of the data they provided so that to motivate them in keep joining a MCS campaign and to reduce their drop-off. 

## Figures and Tables

**Table 1:** An overview of research publications with the description of game involved.

Reference	Type of Game	Co-Operation	Information	Pros	Cons
[97]	Bayesian game	Non-co-operative	Incomplete	Considered asymmetric users	Arbitrary selection with allpay auction
[98]	Repeated game	Non-co-operative	Incomplete	Recruitment of specific task expertees	Requestors forced to pay for poor service
[99]	Coalition game	Yes	Complete	Energy, cost effective sensing	Truthfulness in reputation is left as future work
[100]	Co-operative game between-user and platform	Yes	Incomplete	Budget depend on quality of data obtained	Assumes every device have equal sensing capabilities
[101]	Thesus (based on Bayesian-Nash equilibrium)	Non-co-operative	Complete and Incomplete	Incentives are based on effort, truthfulness	Believed that efforts exerted by user is same for every task
[102]	CSRS (Non-co-operative game)	Non co-operative	Complete	Adaptable for crowded tasks	Believed transmission cost is same for all
[103]	Stackelberg Game	Non-co-operative	Complete and Incomplete	Maximum User Utility on par with Platform	Assumes all users target for longterm gains
[104]	Stackelberg Game	Non-co-operative	Incomplete	Uses WiFi to reduce cost	Not considered user trustworthiness
[105]	Evolutionary game	Non-co-operative	Incomplete	Examined dynamic nature of users	Data reporting delay may occur due late convergence
[106]	Stackelberg game	Yes	Complete and Incomplete	Effective online incentive mechanisms	Considered sensing cost for any task is equal
[107]	Stackelberg game	Yes	Complete, Symmetrically, Asymmetrically Incomplete	Various Incentive mechanisms for CrowdSensing, computing	Not considered negligence of users while sensing the information
[108]	One-shot repeated game	Yes	Complete	Low sensing cost with adequate users	All users in sensing campaign receive part of payments
[109]	Multi-leader Stackelberg game	Non-co-operative	Incomplete	Analyzes pricing competition between crowd sourcers	Not considered resource variation among crowd sourcers
[110]	Stackelberg Bayesian game	Non-co-operative	Complete and Incomplete	Promotes early contributions	Agressive payments
[111]	Three stage stackelberg game	Yes	Complete	Promotes co-operation with low cost	Relies largely on social relationships of users
